# Supplementary material for: The CLASSY family controls tissue-specific DNA methylation patterns in Arabidopsis
Source: Nat Commun. 2022 Jan 11;13:244. doi: 10.1038/s41467-021-27690-x (PMC8752594; doi:10.1038/s41467-021-27690-x)
Supplement: Supplementary file 1 — Supplementary Information [file 41467_2021_27690_MOESM1_ESM.pdf]

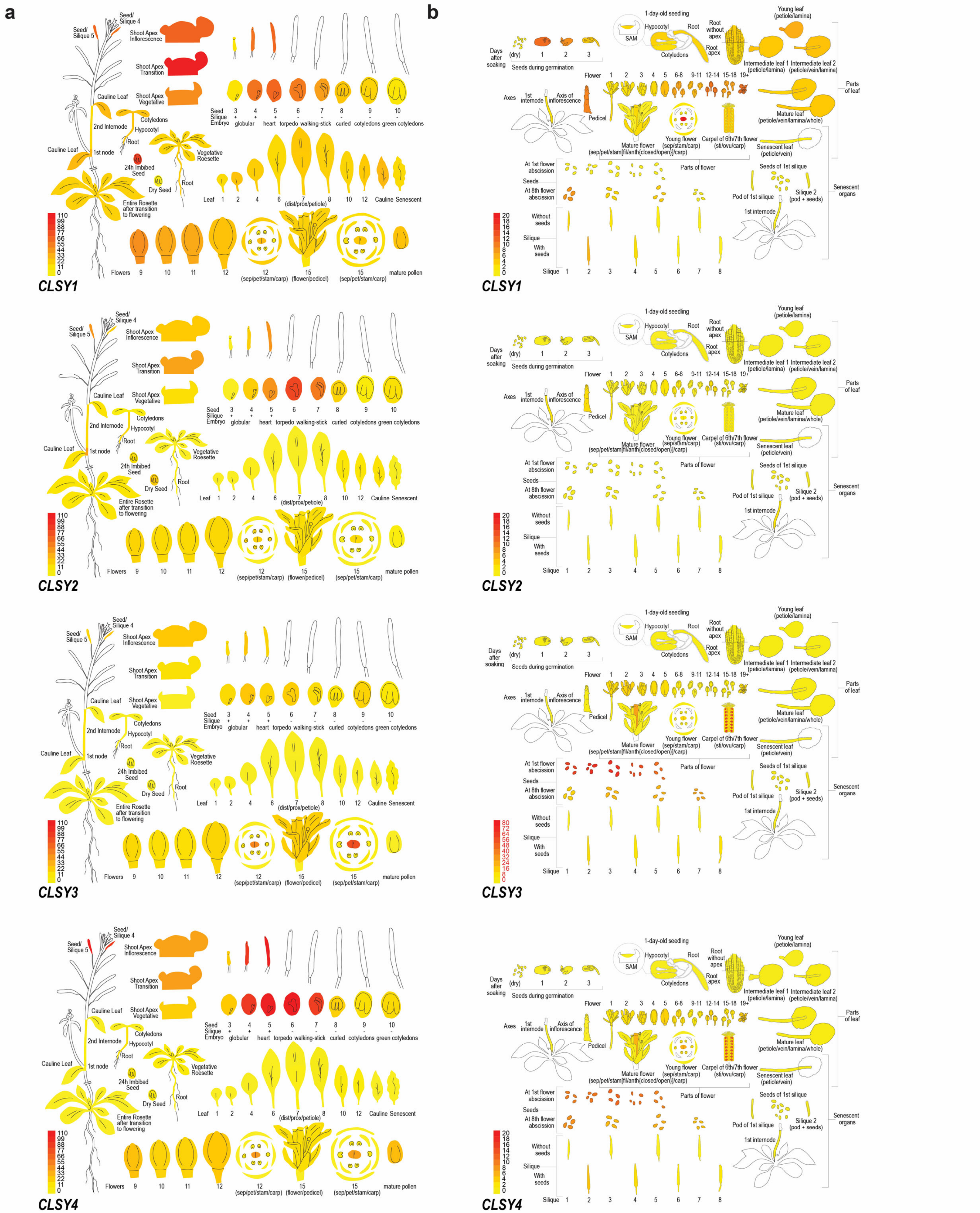

Supplementary Figure 1. Visualization of *CLSY* expression patterns using ePlant[38]. Relative expression levels of the four *CLSY* genes from two Plant eFP viewers: (**a**) AtGenExpress[72, 73] and (**b**) RNA-seq[71]. For comparison, the color scales are equal for panels **a** and **b** with the exception of *CLSY3*, which is on a higher scale, as indicated by the red text on the color bar. All tissues and organs are labeled as in the ePlant viewers[38]. Images are not to scale.

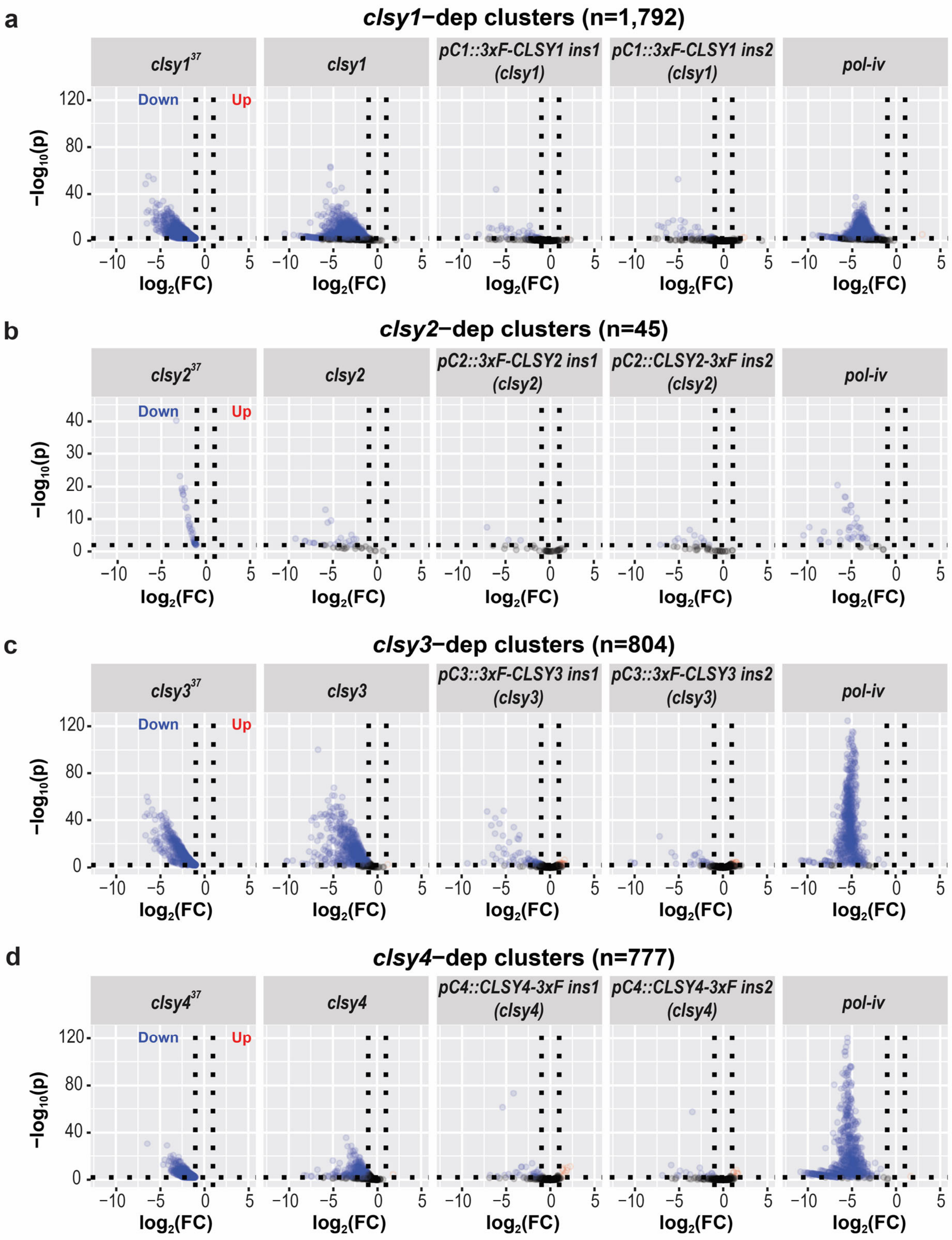

Supplementary Figure 2. Complementation of *clsy* mutants with FLAG-tagged *CLSY* genes. (**a-d**) Volcano plots showing 24nt-siRNA levels at *clsy1*-, *clsy2*-, *clsy3*-, and *clsy4*-dependent clusters (n=1,792, 45, 804, and 777, respectively), as defined in Zhou *et al*. (2018)[37]. For each plot, clusters that are downregulated compared to wild-type controls ($\log_2$ FC ≤ -1 and FDR ≤ 0.01) are shown as blue circles, those unaffected are shown as black circles, and those upregulated ($\log_2$ FC ≥ 1 and FDR ≤ 0.01) are shown as red circles. For all panels, the leftmost plots show previously published data from the indicated *clsy* mutants. The remaining plots show 24nt-siRNA levels in *clsy* and *pol-iv* mutants assayed in parallel with *clsy* mutants complemented with 3xFLAG (3xF)-tagged CLSY constructs. In each case, the *CLSY* genes are driven by their endogenous promoters (pC#) and data are shown from two independent insertion events (ins1 and ins2).

a

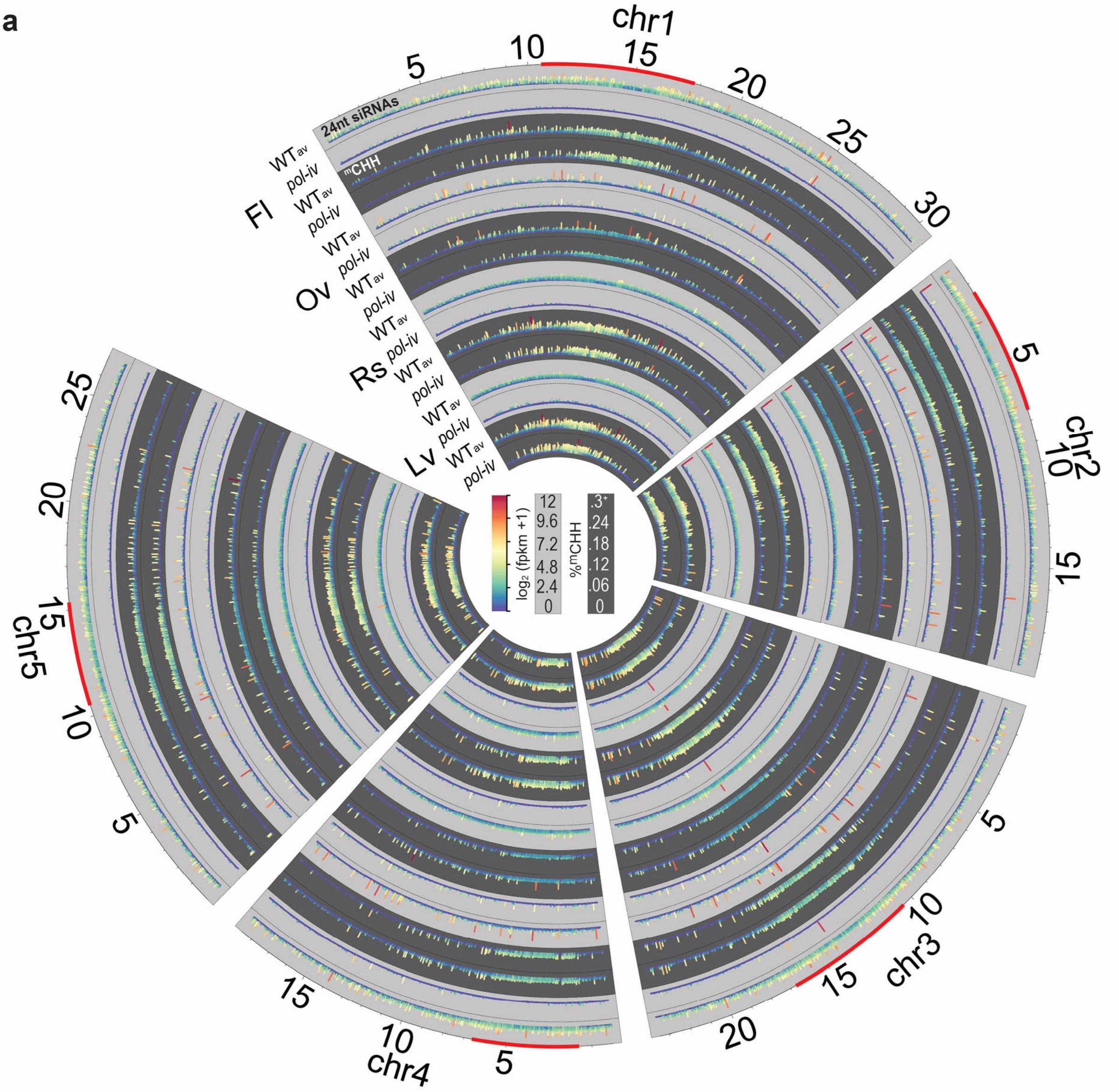

Supplementary Figure 3. Global characterization of 24nt-siRNA and CHH methylation patterns across tissues. (**a**) Circular genome view showing the patterns of 24nt-siRNAs (light grey background) and CHH methylation (dark grey background) across all five chromosomes (chr1-5) in 5kb bins based on the WT_{av} expression levels from each tissue. The color scales for the data sets are as indicated in the center of the plot and the tracks are labeled every 5Mbs, with the pericentromeric heterochromatin, as designated in Yelina *et al.*[69], marked in red along the outer circle. For CHH methylation, between 1 and 3 bins, depending on the tissue, had values over 0.3 (0.3^{+}), but were capped at this value to facilitate visualization on a genomic scale.

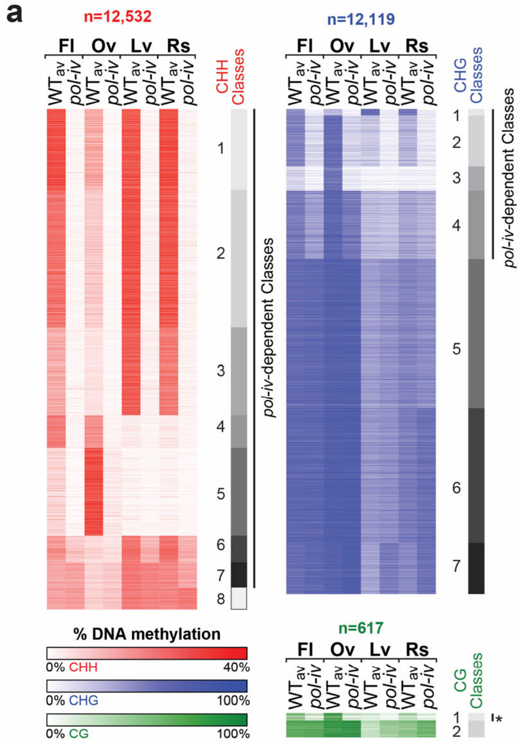

Supplementary Figure 4. Tissue-specific comparison of DNA methylation patterns. (**a**) Scaled heatmaps showing the designation of 8 CHH, 7 CHG, and 2 CG classes of hypo DMRs, respectively, based on the methylation levels (WT_{av} and *pol-iv*) for each tissue at the master set of WTvsWT DMRs for each context (**Supplementary Data 7**). Classes showing clear reductions in methylation in the *pol-iv* mutant are indicated as *pol-iv*-dependent. For CG DMRs, this is indicated by an asterisk.

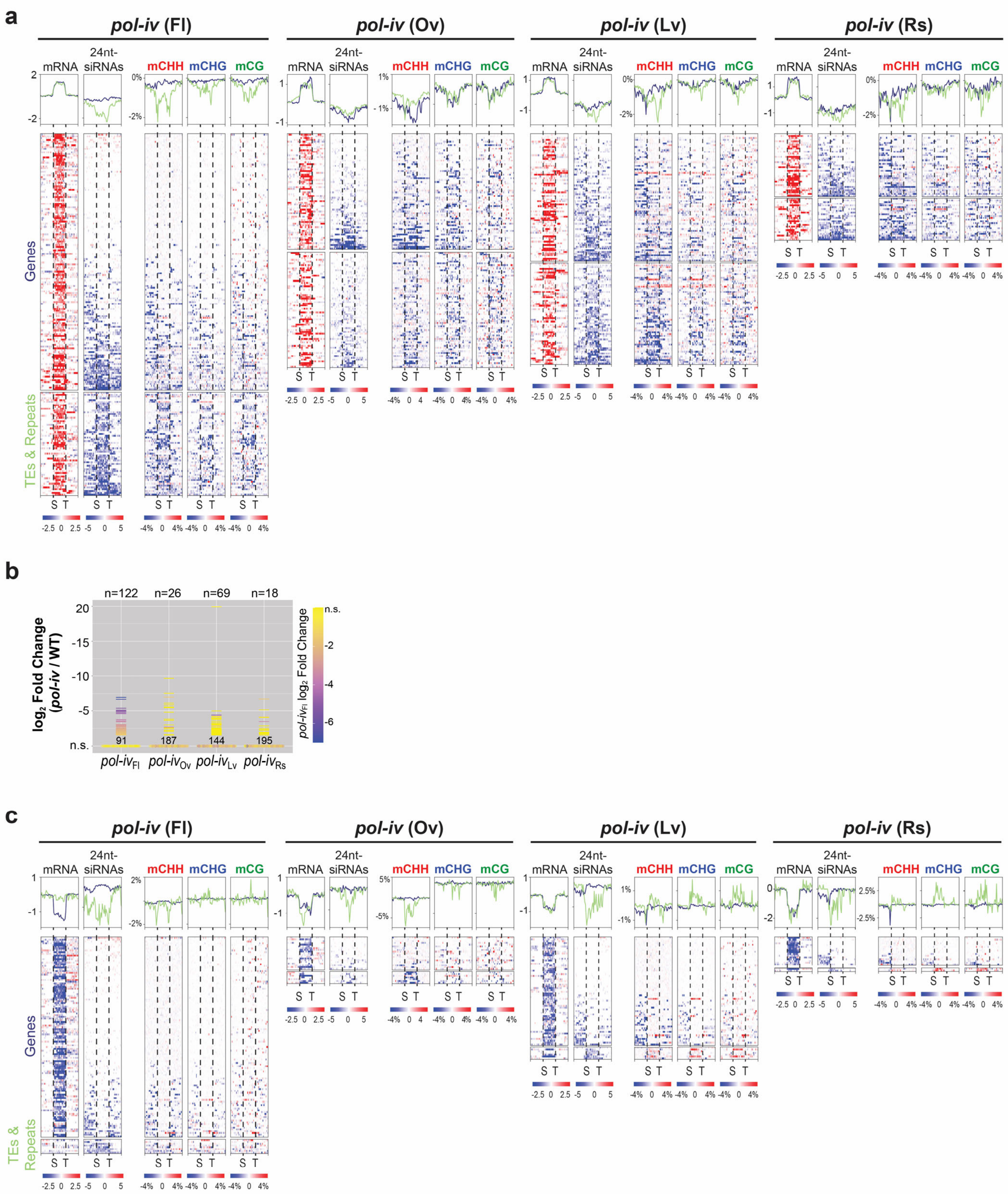

Supplementary Figure 5. Assessment of 24nt-siRNA and DNA methylation levels surrounding differentially expressed transcripts. (**a and c**) Heatmaps and profile plots showing the expression of all *pol-iv* upregulated transcripts (log_2 FC ≥ 1 and FDR ≤ 0.05; as shown in (**Fig. 2g**)) or downregulated transcripts (log_2 FC ≤ -1.5 and FDR ≤ 0.05; as shown in (**Supplementary Fig. 5b**)), respectively, as well as the corresponding 24nt-siRNA and DNA methylation levels at these same loci. For the mRNA and 24nt-siRNA analyses, the log_2 fold changes in expression in *pol-iv* mutants relative to wild-type controls are plotted and for the DNA methylation analysis, the difference in the percent methylation between *pol-iv* mutants and wild-type controls is plotted. Color bars indicating the scales are shown below. The heatmaps include 2kb flanking the transcription start site (S) and the transcription termination site (T) and were ranked based on the 24nt-siRNA and mCHH values for each tissue. The profiles for the genes or TEs/repeats are shown in blue and light green, respectively, above each heatmap. For flower tissue, the data from Zhou *et al*.[37] was reanalyzed using an updated genome annotation (**Supplementary Data 16**). (**b**) Plot showing the expression levels [log_2 FC (*pol-iv*/WT)] of the 213 *pol-iv*-downregulated transcripts represented as horizontal lines (log_2 FC ≤ -1.5 and FDR ≤ 0.05) or dots (not significantly downregulated; n.s.) in the four tissues. The horizontal lines are colored based on the expression level in *pol-iv* from flower tissue (Fl) as shown on the right.

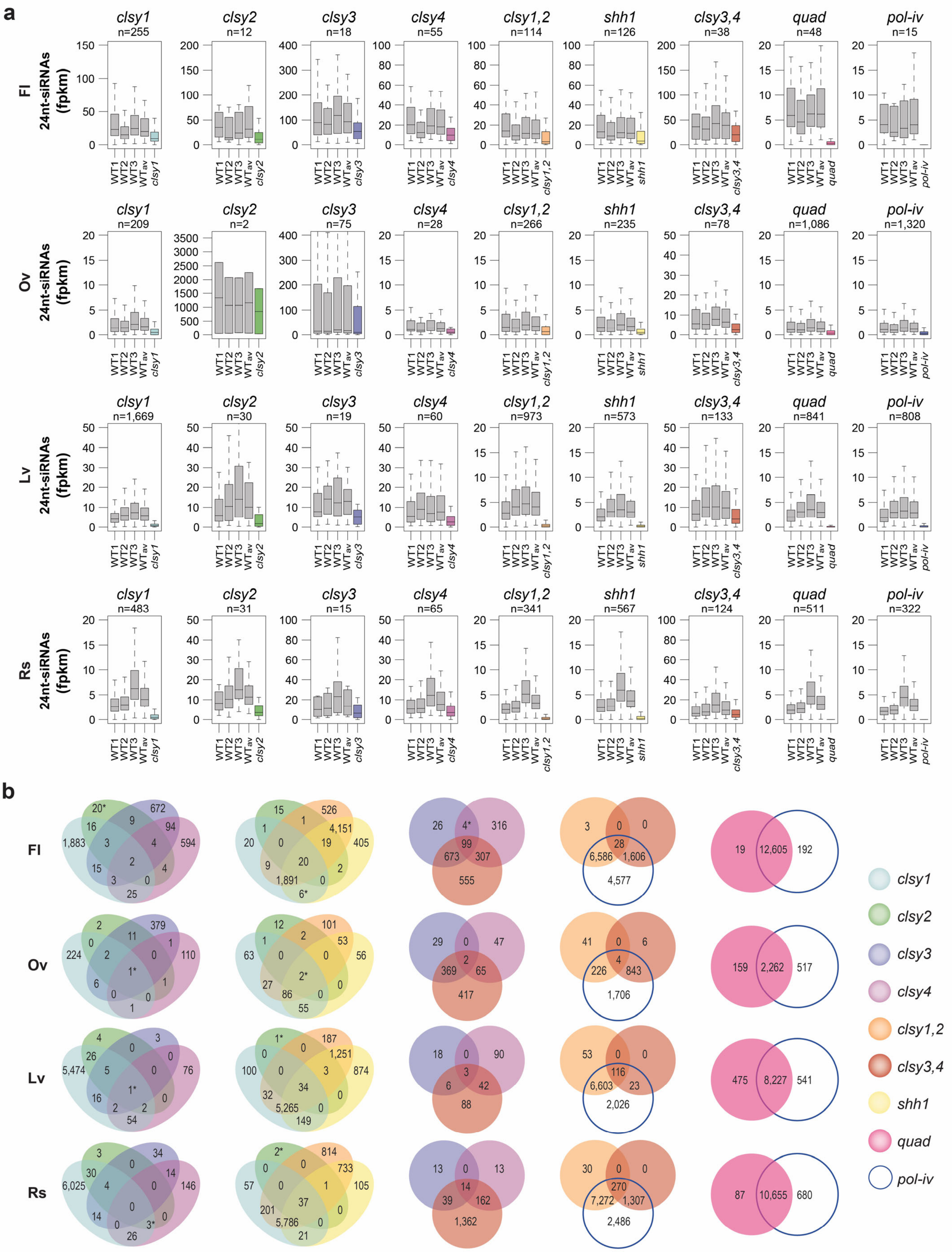

Supplementary Figure 6. Additional analysis of 24nt-siRNA clusters affected in the different tissues and mutant backgrounds. (**a**) Boxplots showing 24nt-siRNA levels at clusters for each tissue and genotype that overlap with hypo CHH DMR(s) and are reduced (>25%) compared to tissue-matched WT controls, but were not initially detected as DE clusters due to the FC and p-value thresholds. Above each plot, the numbers (n) of clusters are indicated and biological replicates for the WT controls are designated as WT1, WT2, and WT3, with the average signal from these replicates designated as the WT_{av}. (**b**) Unscaled Venn diagrams showing the overlaps between 24nt-siRNA clusters affected in each mutant combination and tissue type. Mutants are colored as indicated on the right. * indicates overlaps not included in the scaled Venn diagrams due to spatial constraints.

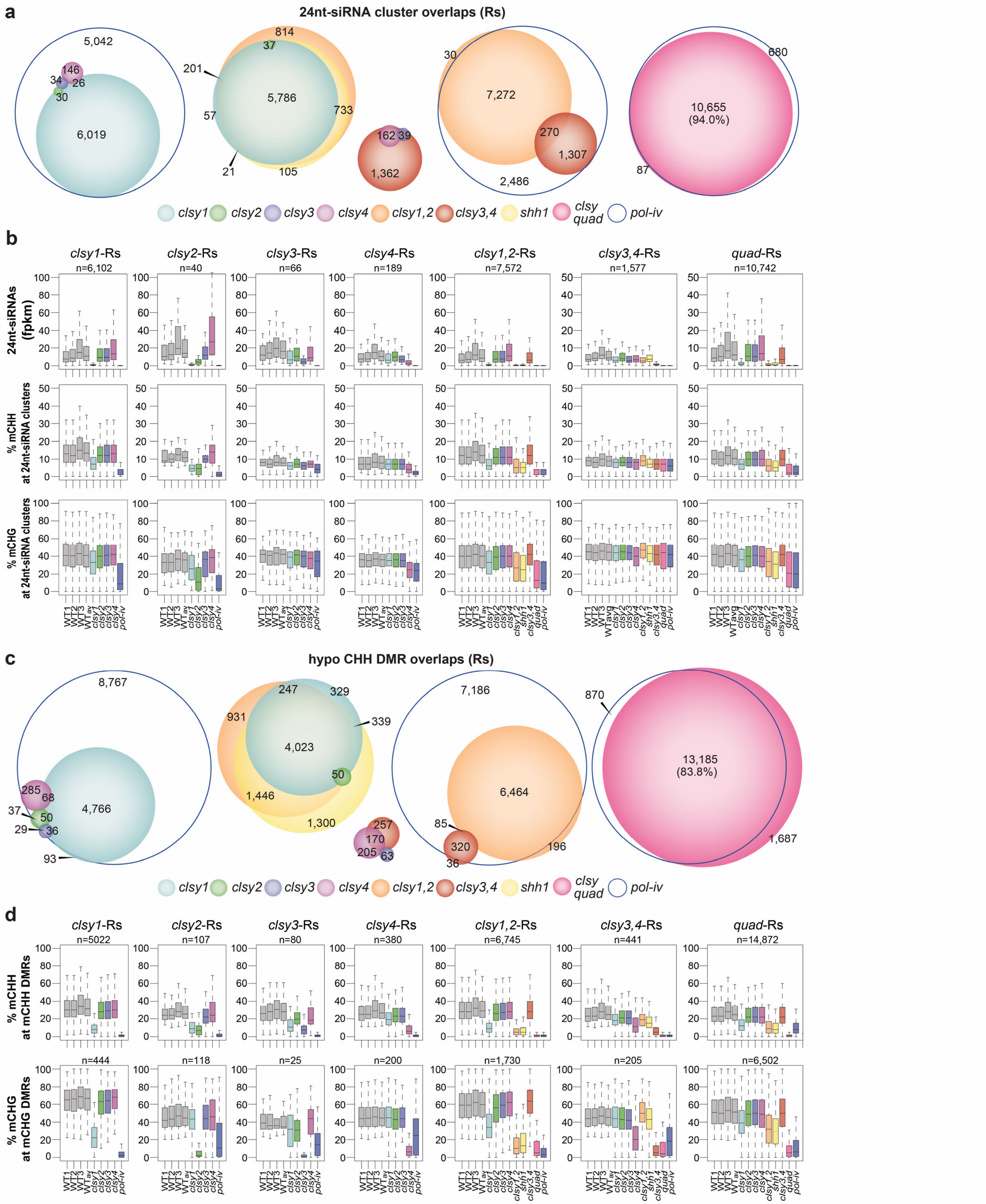

Supplementary Figure 7. Relationships between 24nt-siRNA and DNA methylation levels for *clsy* mutants in rosette (Rs) tissue. (**a** and **c**) Scaled Venn diagrams based on the reduced 24nt-siRNA clusters provided in **Supplementary Data 11** or the hypo CHH DMRs provided in **Supplementary Data 12** showing the relationships between loci affected in the indicated single, double, and quadruple mutants relative to *pol-iv*. For readability, only overlaps >20 are labeled. A small number of overlaps are not shown due to spatial constraints, but unscaled Venn diagrams showing all the overlaps are present in **Supplementary Fig. 6b**. For the 24nt-siRNA clusters, the Venn diagrams for the single and double mutants are also shown in **Fig. 3c**. (**b** and **d**) Boxplots showing 24nt-siRNA and DNA methylation levels at reduced 24nt-siRNA clusters (**b**) or showing DNA methylation levels at hypo CHH and CHG DMRs (**d**). Above each plot, the numbers (n) of reduced 24nt-siRNA clusters identified for each mutant are indicated and biological replicates for the WT controls are designated as WT1, WT2, and WT3, with the average signal from these replicates designated as the WT_{av}.

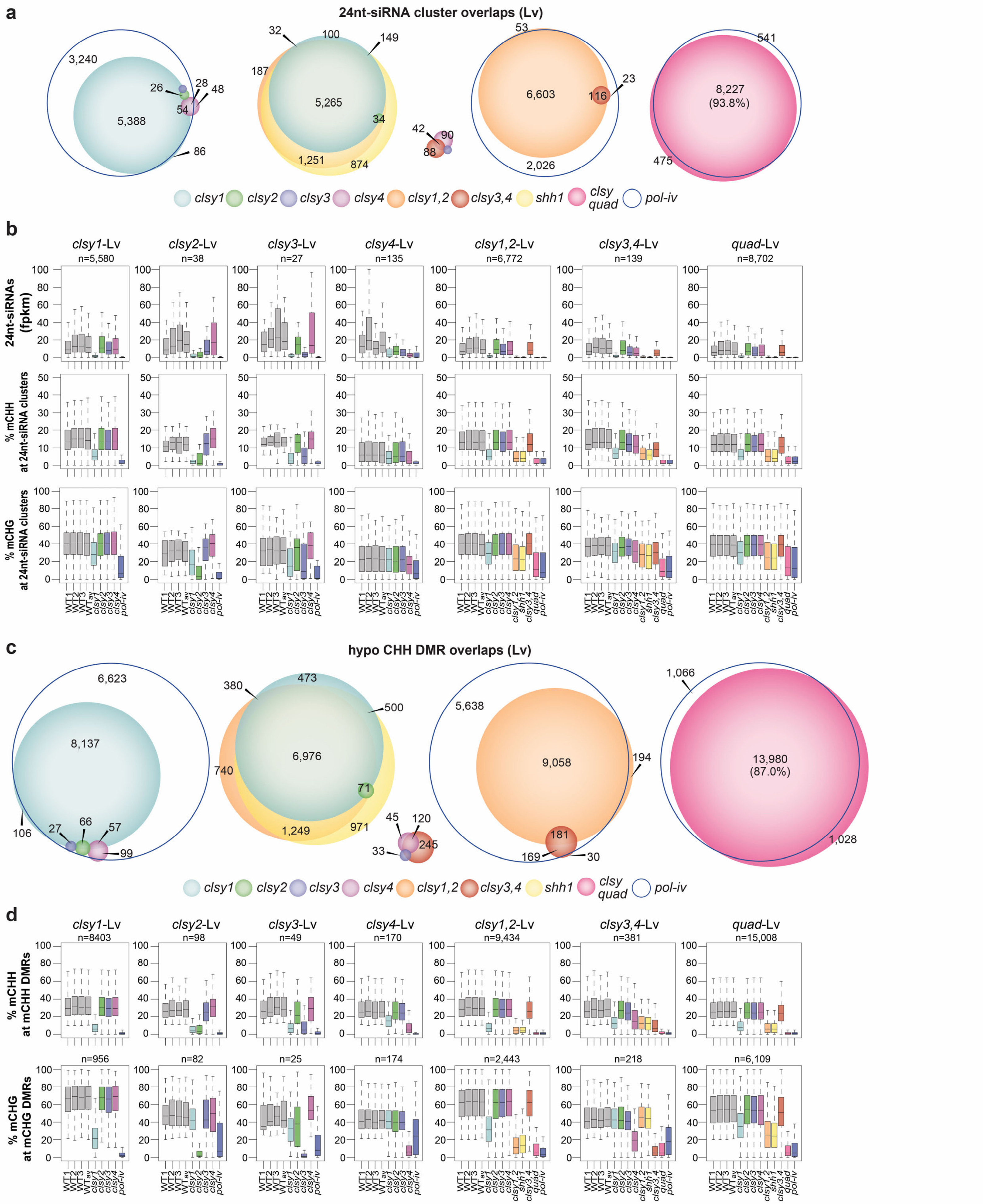

Supplementary Figure 8. Relationships between 24nt-siRNA and DNA methylation levels for *clsy* mutants in leaf (Lv) tissue. (**a** and **c**) Scaled Venn diagrams based on the reduced 24nt-siRNA clusters provided in **Supplementary Data 11** or the hypo CHH DMRs provided in **Supplementary Data 12** showing the relationships between loci affected in the indicated single, double, and quadruple mutants relative to *pol-iv*. For readability, only overlaps >20 are labeled. A small number of overlaps are not shown due to spatial constraints, but unscaled Venn diagrams showing all the overlaps are present in **Supplementary Fig. 6b**. For the 24nt-siRNA clusters, the Venn diagrams for the single and double mutants are also shown in **Fig. 3c**. (**b** and **d**) Boxplots showing 24nt-siRNA and DNA methylation levels at reduced 24nt-siRNA clusters (**b**) or showing DNA methylation levels at hypo CHH and CHG DMRs (**d**). Above each plot, the numbers (n) of reduced 24nt-siRNA clusters identified for each mutant are indicated and biological replicates for the WT controls are designated as WT1, WT2, and WT3, with the average signal from these replicates designated as the WT_{av}.

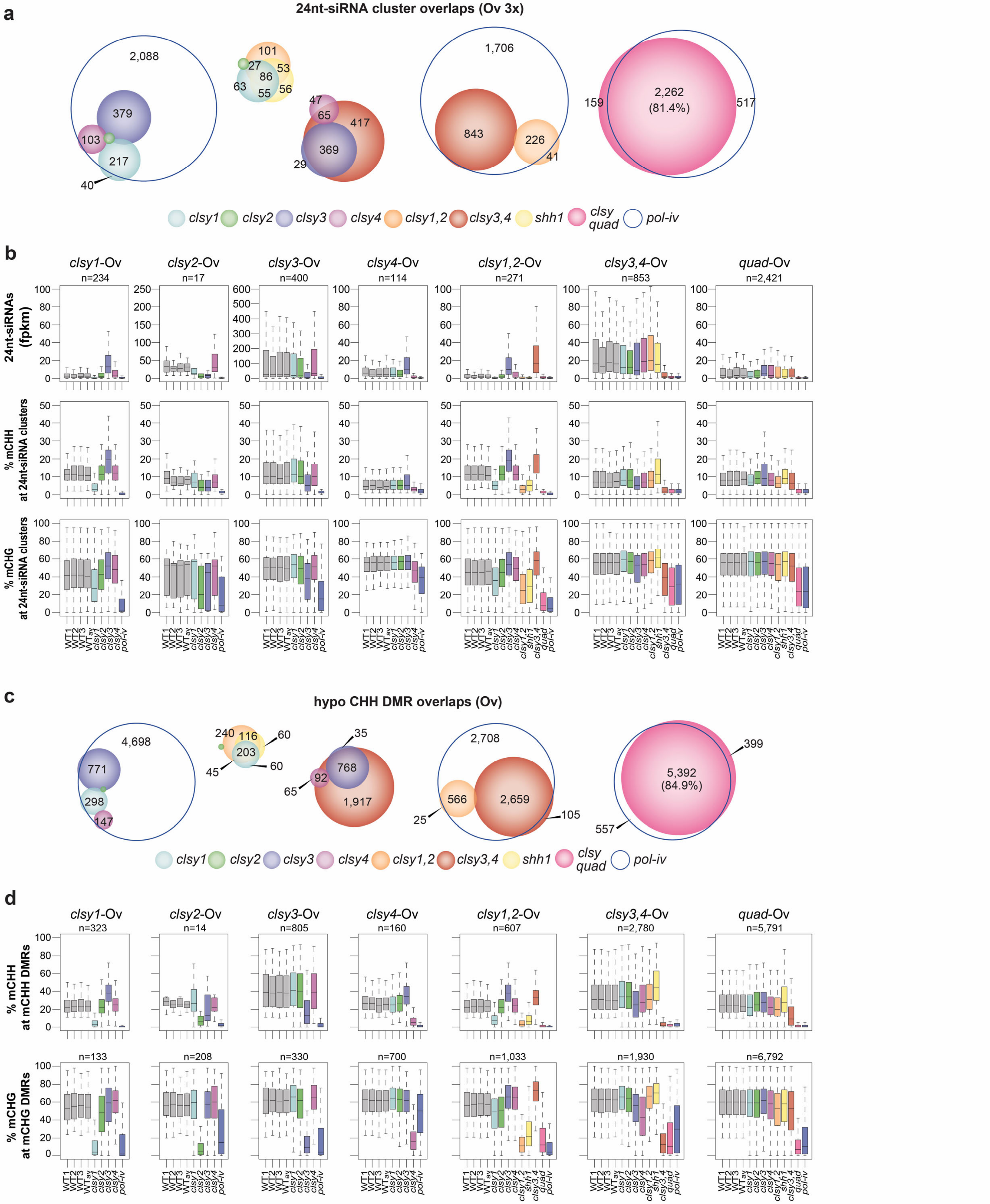

Supplementary Figure 9. Relationships between 24nt-siRNA and DNA methylation levels for *clsy* mutants in ovule (Ov) tissue. (**a** and **c**) Scaled Venn diagrams based on the reduced 24nt-siRNA clusters provided in **Supplementary Data 11** or the hypo CHH DMRs provided in **Supplementary Data 12** showing the relationships between loci affected in the indicated single, double, and quadruple mutants relative to *pol-iv*. For readability, only overlaps >20 are labeled. A small number of overlaps are not shown due to spatial constraints, but unscaled Venn diagrams showing all the overlaps are present in **Supplementary Fig. 6b**. For the 24nt-siRNA clusters, the Venn diagrams for the single and double mutants are also shown in **Fig. 3c.** (**b** and **d**) Boxplots showing 24nt-siRNA and DNA methylation levels at reduced 24nt-siRNA clusters (**b**) or showing DNA methylation levels at hypo CHH and CHG DMRs (**d**). Above each plot, the numbers (n) of reduced 24nt-siRNA clusters identified for each mutant are indicated and biological replicates for the WT controls are designated as WT1, WT2, and WT3, with the average signal from these replicates designated as the WT_{av}.

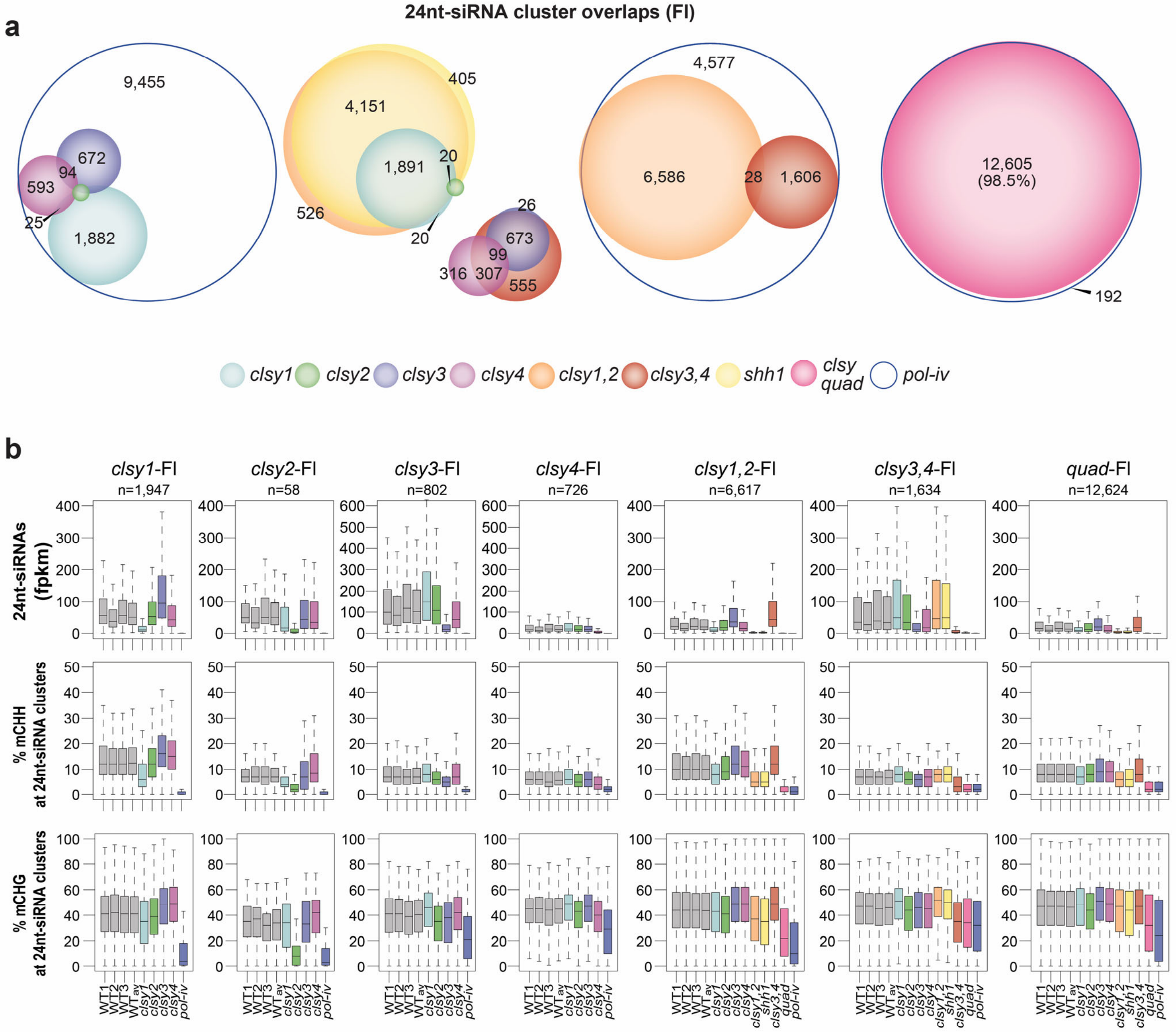

Supplementary Figure 10. Relationships between 24nt-siRNA and DNA methylation levels for *clsy* mutants in flower (Fl) tissue. (**a**) Scaled Venn diagrams based on the reduced 24nt-siRNA clusters provided in **Supplementary Data 11** showing the relationships between loci affected in the indicated single, double, and quadruple mutants relative to *pol-iv* mutants. For readability, only overlaps >20 are labeled. A small number of overlaps are not shown due to spatial constraints, but unscaled Venn diagrams showing all the overlaps are present in **Supplementary Fig. 6b**. For the 24nt-siRNA clusters, the Venn diagrams for the single and double mutants are also shown in **Fig. 3c**. (**b**) Boxplots showing 24nt-siRNA and DNA methylation levels at reduced 24nt-siRNA clusters for the *clsy* mutants indicated above each set of plots. Above each plot, the numbers (n) of reduced 24nt-siRNA clusters identified for each mutant are indicated and biological replicates for the WT controls are designated as WT1, WT2, and WT3, with the average signal from these replicates designated as the WT_{av}. Methylation levels for hypo CHH and CHG DMRs in the *clsy* and *pol-iv* mutants are published in Zhou *et al*.[37].

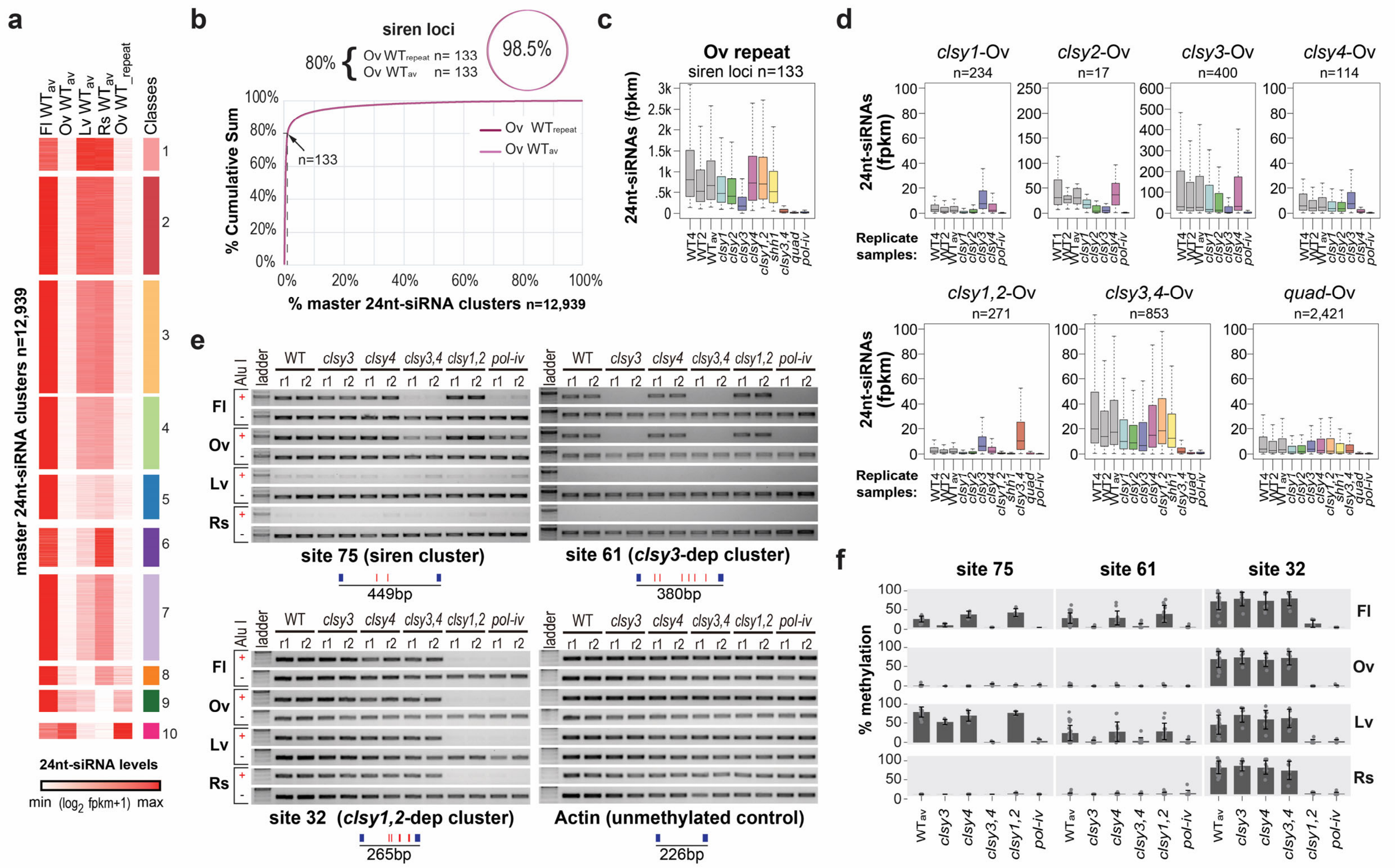

Supplementary Figure 11. Analysis of 24nt-siRNA and DNA methylation patterns shows consistent behavior between replicates. (**a**) Heatmap, clustered as in **Fig. 2c**, showing the expression levels of 24nt-siRNAs from the initial set of WT tissues (Fl WT_{av}, Ov WT_{av}, Lv WT_{av}, and Rs Wt_{av}) and from an ovule replicate (Ov WT_repeat). (**b**) Cumulative sum plot showing the percentage and number (n) of 24nt-siRNA clusters (x-axis) required to reach 80% of the fpkm-normalized 24nt-siRNA reads (y-axis) from the initial ovule data (Ov WT_{av}) and from an ovule replicate (Ov WT_repeat). The Venn diagram (at 1.5x scale) shows a nearly perfect overlap between the 133 siren loci identified between replicates. (**c** and **d**) Boxplots showing 24nt-siRNA levels from a repeat set of ovule data (WT and all *clsy* mutant combinations) at either the initially defined siren loci (**c**) or the initially defined reduced 24nt-siRNA clusters for each *clsy* mutant (**d**). Above each plot, the numbers (n) of reduced 24nt-siRNA clusters identified for each mutant are indicated and biological replicates for the WT controls are designated as WT2 and WT4 with the average signal from these replicates designated as the WT_{av}. (**e**) Methyl-cutting assays showing the amplification of four regions using DNA from two independent replicates (r1 and r2) from the indicated tissues (left) and genotypes (top) after digestion with the methylation-sensitive AluI restriction enzyme (AluI +) or a mock treatment (AluI -). In the AluI + samples, lower signal corresponds to reductions in DNA methylation. The AluI - samples, and amplification at the *Actin* locus, which lacks AluI sites, serve as controls. This experiment was conducted once and the regions corresponding the PCR primers and AluI restriction sites are indicated below in blue and red, respectively, with the sizes indicated in bps. (**f**) Plots showing the individual methylation levels (dots) and average percent methylation (bars) of the cytosines within the AluI sites shown in panel **e**. The data is based on the MethylC-seq data from the indicated tissues (right) and genotypes (bottom). In the WT_{av} samples, the data includes three replicates (WT1, WT2, and WT3), while the other genotypes are from a single dataset. All error bars represent the standard deviation.

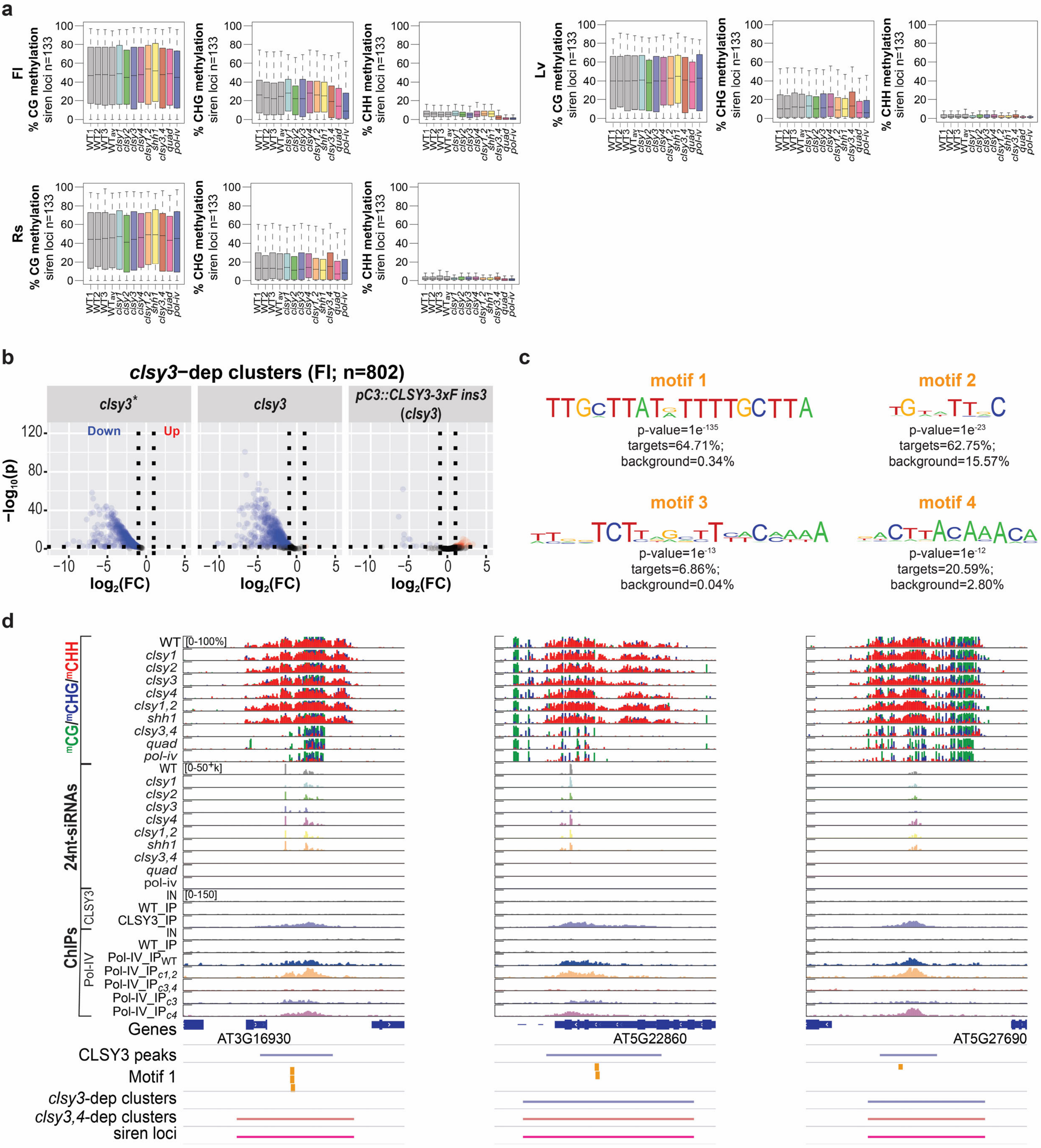

Supplementary Figure 12. Assessment of DNA methylation levels at siren loci and motifs under CLSY3 peaks. (**a**) Boxplots showing DNA methylation levels at siren 24nt-siRNA clusters (n=133) in the indicated genotypes and tissues. (**b**) Volcano plots showing 24nt-siRNA levels at *clsy3*-dependent clusters identified from flower tissue (n=802). For each plot, clusters that are downregulated compared to wild-type controls ($\log_2$ FC ≤ -1 and FDR ≤ 0.01) are shown as blue circles, those unaffected are shown as black circles, and those upregulated ($\log_2$ FC ≥ 1 and FDR ≤ 0.01) are shown as red circles. The left plot shows 24nt-siRNA data from the *clsy3* mutant sample characterized in **Fig. 3**, denoted as *clsy3**. The adjacent plots show data from an independent *clsy3* sample assayed in parallel with a *clsy3* mutant complemented with a 3xFLAG (3xF)-tagged CLSY3 construct driven by its endogenous promoter (pC3) (**c**) Top four motifs identified from the CLSY3 ChIP, including their p-values and the percentage of each motif found in target regions (CLSY3 peaks n=102) or background regions (TAIR10 genome n=43,700). (**d**) Screenshots showing the levels of DNA methylation (% methylation in each context), 24nt-siRNAs, and CLSY3 or Pol-IV enrichment from flower tissue in the indicated genotypes at representative CLSY3 peaks. Track scales are the same for all three sites and are indicated in brackets. The regions corresponding to different features are indicated below. For the Pol-IV ChIP data the IP subscripts indicate the genetic background (*e.g.* IP_{c3} indicates the *clsy3* background).

a

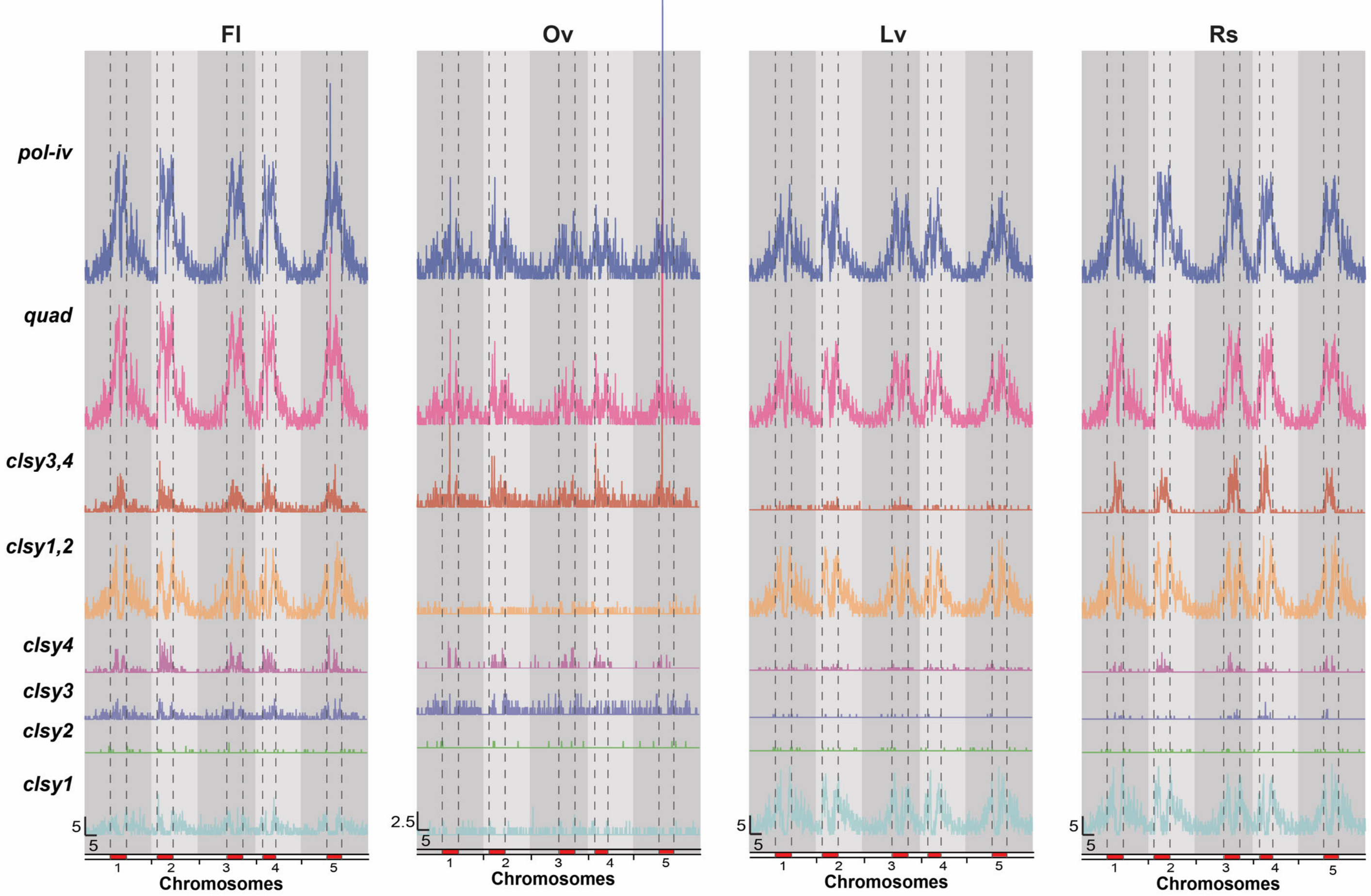

Supplementary Figure 13. Chromosomal Distributions of 24nt-siRNA clusters. (**a**) Distributions of reduced 24nt-siRNA clusters, in the indicated genotypes and tissues, along the 5 chromosomes. The pericentromeric heterochromatin, as designated in Yelina *et al*.[69], is marked in red and denoted by vertical dashed lines. Scale bars for x-axis (Mb) and y-axis (clusters/100kb bin) are indicated in the lower left corner of each set. Note that ovules are at 2x relative to the other tissues. Data for the *clsy1,2* and *clsy3,4* clusters are also shown in **Fig. 5e**.

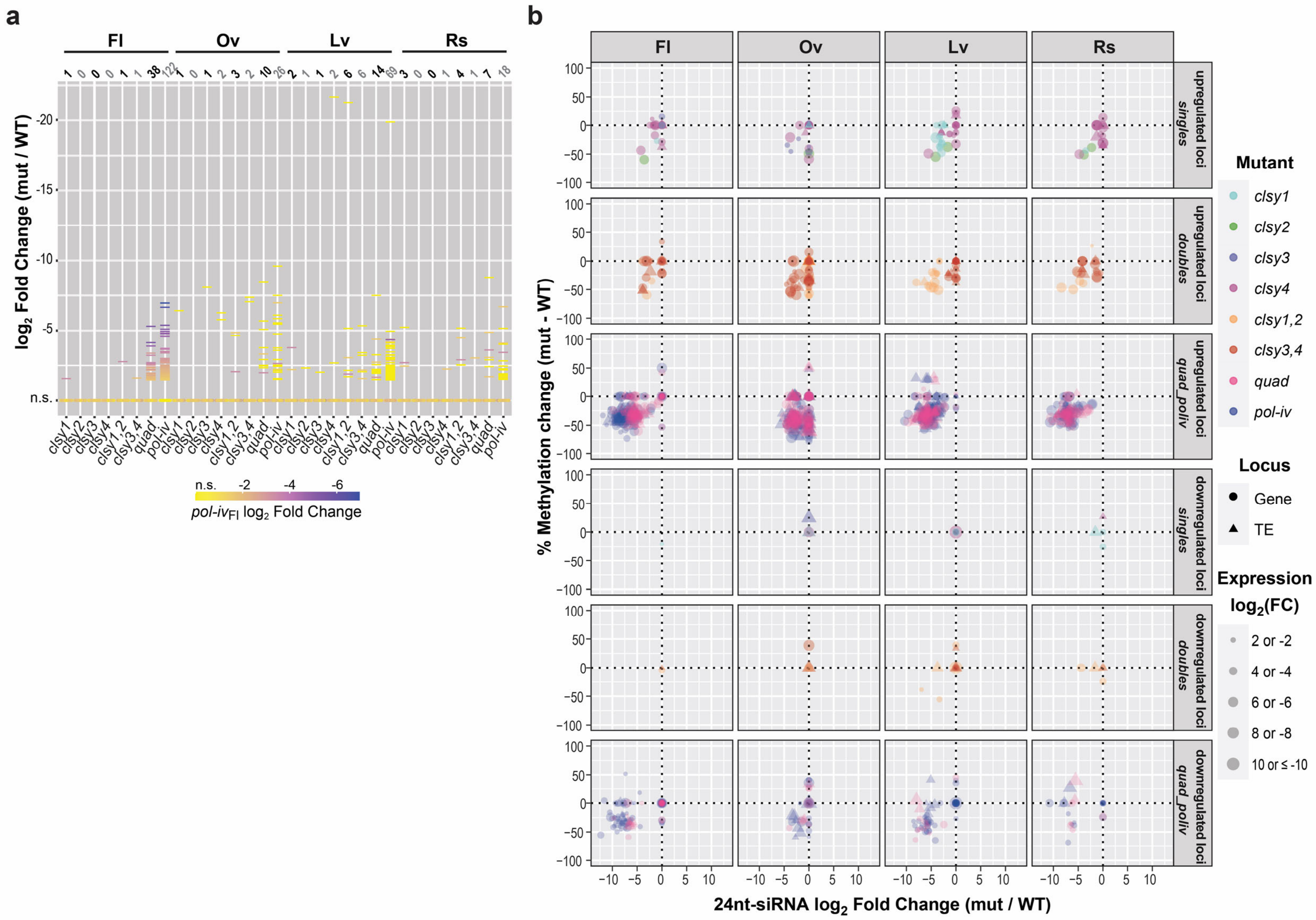

Supplementary Figure 14. Association of DE loci with changes in epigenetic features. (**a**) Plot showing the expression levels [log_2 FC (mut/WT)] of the 213 *pol-iv*-downregulated transcripts represented as horizontal lines (log_2 FC ≤ -1.5 and FDR ≤ 0.05) or as dots (not significantly downregulated; n.s.) in the four tissues. The horizontal lines are colored based on the expression level in *pol-iv* from flower tissue and the number of DE loci are indicated above each column. (**b**) Bubble plots showing the expression changes of genes (circles) or TEs (triangles) in the mutants and tissues indicated, as well as the changes in 24nt-siRNAs and nonCG methylation at these loci and the surrounding regions (+/- 2Kb). For each locus, the average log2 fold change in expression across all overlapping reduced 24-nt siRNA clusters is plotted on the x-axis [Average (log2 FC mut/WT)] and the average difference in methylation levels across all overlapping DMRs is plotted on the y-axis [Average (% methylation mut – WT)].

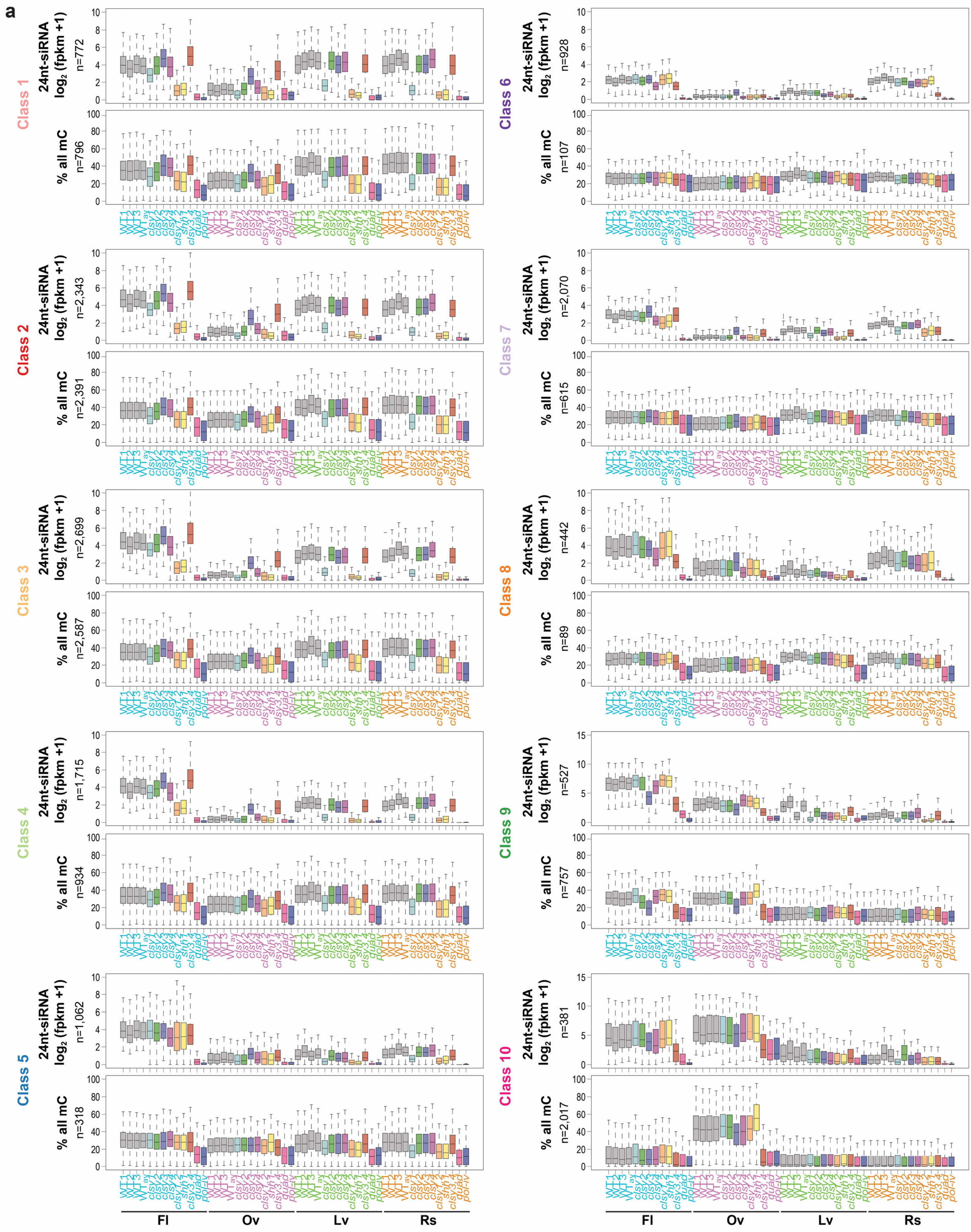

Supplementary Figure 15. 24nt-siRNA and DNA methylation levels in relation to the 10 24nt-siRNA classes. (**a**) Boxplots showing 24nt-siRNA and DNA methylation levels in all sequence contexts (% all mC) at reduced 24nt-siRNA clusters grouped by the ten classes defined in **Fig. 2c** (upper) or at hypo CHH DMRs within these clusters (lower) for the genotypes and tissues indicated below. The numbers (n) of clusters or DMRs are indicated and biological replicates for the WT controls are designated as WT1, WT2, and WT3, with the average signal from these replicates designated as the WT_{av}.
