# Supplementary material for: The CLASSY family controls tissue-specific DNA methylation patterns in Arabidopsis
Source: Nat Commun. 2022 Jan 11;13:244. doi: 10.1038/s41467-021-27690-x (PMC8752594; doi:10.1038/s41467-021-27690-x)
Supplement: Supplementary file 2 — Description of Additional Supplementary Files [file 41467_2021_27690_MOESM2_ESM.docx]

Description of Additional Supplementary Files

File name: Supplementary Data 1

Description: Summary of mRNA-seq data

File name: Supplementary Data 2

Description: Summary of smRNA-seq data

File name: Supplementary Data 3

Description: Summary of MethylC-seq data

File name: Supplementary Data 4

Description: 21-24nt small RNA clusters

File name: Supplementary Data 5

Description: 24nt-siRNA cluster lists and summary

File name: Supplementary Data 6

Description: 24nt-siRNA heatmap clustering and summary

File name: Supplementary Data 7

Description: DMRs between WT tissues and summary

File name: Supplementary Data 8

Description: DNA methylation heatmap clustering and summary

File name: Supplementary Data 9

Description: clsy DEGs overlapping with pol-iv and summary

File name: Supplementary Data 10

Description: pol-iv DE analysis and summary

File name: Supplementary Data 11

Description: DE 24nt-siRNA clusters and summary

File name: Supplementary Data 12

Description: hypo DMRs and summary

File name: Supplementary Data 13

Description: Summary of ChIP-seq data

File name: Supplementary Data 14

Description: ChIP analysis and summary

File name: Supplementary Data 15

Description: Primers table

File name: Supplementary Data 16

Description: GFF file
