# Supplementary material for: The CLASSY family controls tissue-specific DNA methylation patterns in Arabidopsis
Source: Nat Commun. 2022 Jan 11;13:244. doi: 10.1038/s41467-021-27690-x (PMC8752594; doi:10.1038/s41467-021-27690-x)
Supplement: Supplementary file 19 — Reporting Summary [file 41467_2021_27690_MOESM19_ESM.pdf]

nature portfolio

Corresponding author(s): Julie A. Law

Last updated by author(s): Dec 4, 2021

Reporting Summary

Nature Portfolio wishes to improve the reproducibility of the work that we publish. This form provides structure for consistency and transparency in reporting. For further information on Nature Portfolio policies, see our Editorial Policies and the Editorial Policy Checklist.

Statistics

For all statistical analyses, confirm that the following items are present in the figure legend, table legend, main text, or Methods section.

n/a	Confirmed	
☐	☒	The exact sample size (n) for each experimental group/condition, given as a discrete number and unit of measurement
☐	☒	A statement on whether measurements were taken from distinct samples or whether the same sample was measured repeatedly
☐	☒	The statistical test(s) used AND whether they are one- or two-sided<br>*Only common tests should be described solely by name; describe more complex techniques in the Methods section.*
☒	☐	A description of all covariates tested
☒	☐	A description of any assumptions or corrections, such as tests of normality and adjustment for multiple comparisons
☒	☐	A full description of the statistical parameters including central tendency (e.g. means) or other basic estimates (e.g. regression coefficient) AND variation (e.g. standard deviation) or associated estimates of uncertainty (e.g. confidence intervals)
☐	☒	For null hypothesis testing, the test statistic (e.g. F, t, r) with confidence intervals, effect sizes, degrees of freedom and P value noted<br>*Give P values as exact values whenever suitable.*
☒	☐	For Bayesian analysis, information on the choice of priors and Markov chain Monte Carlo settings
☒	☐	For hierarchical and complex designs, identification of the appropriate level for tests and full reporting of outcomes
☒	☐	Estimates of effect sizes (e.g. Cohen's d, Pearson's r), indicating how they were calculated

Our web collection on statistics for biologists contains articles on many of the points above.

Software and code

Policy information about availability of computer code

Data collection	n/a
Data analysis	cutadapt (v1.9.1)<br>ShortStack (v3.8.1)<br>HOMER (v2.4.0)<br>splitTagDirectoryByLength.dev2.pl (a custom perl script)<br>JSON_findPerfectMatches_and_TerminalMisMatches_v3 (a custom JSON script)<br>splitpancakesbysize_shortStack_v3.8.1 (a custom perl script)<br>DESeq2 (v1.30.1)<br>R using Rstudio (v1.4.1106)<br>Morpheus (https://software.broadinstitute.org/morpheus/)<br>VENNY2.1 (http://bioinfogp.cnb.csic.es/tools/venny/)<br>VennMaster (v0.38.2)<br>IGV genome browser (v2.6.3)<br>BS-seeker2 (v2.0.8)<br>remove_clonal.py (a custom python script for calling picard function)<br>picard (v2.16.0)<br>BSseeker2_2_wiggleV2.pl (a custom perl script)<br>BSseeker2_methylCall2Cytosine.pl (a custom perl script)<br>CytosineTo100bpBin.pl (a custom perl script)<br>GetOnlyCommonBins.pl (a custom perl script)<br>DMRFtestFDR.R (a custom R script)

SplittingDMRs.pl (a custom perl script)
SplittingDMR2Bed.pl (a custom perl script)
bedops (v2.4.8)
bedmap (v2.4.8)
parseWig_noChr.v2.pl (a custom perl script)
STAR (v2.5.0c)
ggplot2 (v3.3.5)
bowtie (v1.1.0)
deepTools (v2.4.0)
methylkit (0.9.2)
circlize (0.4.13)

For manuscripts utilizing custom algorithms or software that are central to the research but not yet described in published literature, software must be made available to editors and reviewers. We strongly encourage code deposition in a community repository (e.g. GitHub). See the Nature Portfolio guidelines for submitting code & software for further information.

Data

Policy information about availability of data

All manuscripts must include a data availability statement. This statement should provide the following information, where applicable:

- Accession codes, unique identifiers, or web links for publicly available datasets
- A description of any restrictions on data availability
- For clinical datasets or third party data, please ensure that the statement adheres to our policy

Illumina sequencing data (smRNA-seq, MethylC-seq, mRNA-seq, and ChIP-seq) has been deposited in the NCBI Gene Expression Omnibus (GEO) and are accessible through the GEO series accession number GSE165001.

Field-specific reporting

Please select the one below that is the best fit for your research. If you are not sure, read the appropriate sections before making your selection.

☒ Life sciences ☐ Behavioural & social sciences ☐ Ecological, evolutionary & environmental sciences

For a reference copy of the document with all sections, see nature.com/documents/nr-reporting-summary-flat.pdf

Life sciences study design

All studies must disclose on these points even when the disclosure is negative.

Sample size	no experiments were conducted that required consideration of a sample size.
Data exclusions	The only data excluded was for visualization of CHH methylation across the 5 chromosomes. This is clearly stated in the figure legends. "For CHH methylation, between 1 and 3 bins, depending on the tissue, had values > 0.3 (0.3+), but were capped at 0.3 to facilitate genome-wide visualization"
Replication	mRNA-seq: For each tissue and genotype, two biological replicates are included and used for the DESeq2 analysis.<br>smRNA-seq: For each tissue, mutant samples were compared to three wild-type controls. We also provided an independent replicate for all genotypes from ovule tissue.<br>methylC-seq: For each tissue, mutant samples were compared to three wild-type controls. We also provided an independent replicated of select genotypes for all four tissues at three loci using a methyl-cutting assay.<br><br>In all cases the data replications were successful.
Randomization	No assays were conducted that required randomization.
Blinding	Blinding was not relevant to this study as nearly all analyses were based on illumina sequencing, and thus were not prone to individual/ subjective biases.

Reporting for specific materials, systems and methods

We require information from authors about some types of materials, experimental systems and methods used in many studies. Here, indicate whether each material, system or method listed is relevant to your study. If you are not sure if a list item applies to your research, read the appropriate section before selecting a response.

Materials & experimental systems

n/a	Involved in the study	
☐	☒	Antibodies
☒	☐	Eukaryotic cell lines
☒	☐	Palaeontology and archaeology
☐	☒	Animals and other organisms
☒	☐	Human research participants
☒	☐	Clinical data
☒	☐	Dual use research of concern

Methods

n/a	Involved in the study	
☐	☒	ChIP-seq
☒	☐	Flow cytometry
☒	☐	MRI-based neuroimaging

Antibodies

Antibodies used: anti-Flag M2 Magnetic beads (Sigma, Cat# M8823)

Validation: The anti-Flag M2 Magnetic beads where used for the 3xFLAG tagged CLSY3 ChIP assay. Incubation of the beads with material from wild-type plant material was included as a negative control to account for any non-specific binding of the antibody.

Animals and other organisms

Policy information about studies involving animals; ARRIVE guidelines recommended for reporting animal research

Laboratory animals: No laboratory animals were used

Wild animals: No wild animals were used.

Field-collected samples: No field-collected samples were used.

Ethics oversight: No ethical oversights were required for this study.

Note that full information on the approval of the study protocol must also be provided in the manuscript.

ChIP-seq

Data deposition

☒ Confirm that both raw and final processed data have been deposited in a public database such as GEO.

☒ Confirm that you have deposited or provided access to graph files (e.g. BED files) for the called peaks.

Data access links
May remain private before publication.
https://www.ncbi.nlm.nih.gov/geo/query/acc.cgi?acc=GSE164578

Files in database submission:
GSM5014383_WT_Fl_input.ucsc.bedGraph.gz
GSM5014384_WT_Fl_IP.ucsc.bedGraph.gz
GSM5014385_CLSY3-3xFLAG_Fl_IP.ucsc.bedGraph.gz

Genome browser session (e.g. UCSC): We do not have a publicly available genome browser available, but the tracks above can be loaded into IGV.

Methodology

Replicates: This experiment has just one replicate.

Sequencing depth:
Genotype_ChIP/Tissue/ Uniquely mapped Reads (see also Table S13)
Col_input/ flower buds (stage 12 and younger) / 17,219,108
Col_IP / flower buds (stage 12 and younger) / 21,635,925
CLSY3-3xFLAG_IP / flower buds (stage 12 and younger) / 21,119,935

Antibodies: anti-Flag M2 Magnetic beads (Sigma, Cat# M8823)

Peak calling parameters: The ChIP-seq data was aligned to TAIR10 reference genome using bowtie (v1.1.0) and the "-m 1 -v 2 --all –best and –strata" options to allow 2 mismatches and including only uniquely mapping reads (Supplementary Table 13). TagDirectories and UCSC genome browser tracks from ChIP-seq data were generated with the makeTagDirectory and makeUCSCfile scripts from HOMER using the "format sam -mis 2 and -unique" or "none -fragLength given and -norm 10000000" options, respectively. The 102 CLSY3 ChIP peaks were identified with the HOMER findPeaks script using the "-style factor -region -L 2.5 -F 2.5 and -center" options (see Supplementary Table 14).

Data quality	For peak calling using the findPeaks script, an fdr<=0.001 is the default parameter and our addition of the F2.5 parameter requires a 2.5 fold change compared to the control. Thus all our 102 peaks meet these two criteria.
Software	As detailed above, Bowtie was used for the read mapping and the HOMER findPeaks script was used for peak calling and for the generation of UCSC browser tracks. deepTools (v2.4.0) was used to visualized the data.
